# Graphitic carbon nitride/graphene nanoflakes hybrid system for electrochemical sensing of DNA bases in meat samples

**DOI:** 10.1038/s41598-020-69578-8

**Published:** 2020-07-30

**Authors:** J. Kalaiyarasi, K. Pandian, Santheraleka Ramanathan, Subash C. B. Gopinath

**Affiliations:** 1 Department of Inorganic Chemistry, University of Madras, Guindy campus, Chennai, 600 025 India; 2 Institute of Nano Electronic Engineering, 01000 Kangar, Malaysia; 3 School of Bioprocess Engineering, Universiti of Malaysia Perlis, 02600 Arau, Perlis Malaysia

**Keywords:** Biochemistry, Biotechnology, Materials science, Nanoscience and technology

## Abstract

This research presents a simple, fast and simultaneous electrochemical quantitative determination of nucleobases, for example guanine (G), adenine (A), and thymine (T) in a beef and chicken livers samples to measure the quality of food products based on hybrids of graphitic carbon nitride/Graphene nanoflakes (g-C_3_N_4_/GNF) modified electrode. Graphitic carbon nitride (g-C_3_N_4_) made of graphite-like covalent link connects nitrogen, nitride, and carbon atoms in the structural design with improved the electrical properties and low band gap semiconductor. The g-C_3_N_4_/GNF nanocomposite was synthesized by the hydrothermal treatment to form a porous g-C_3_N_4_ interconnected three dimensional (3D) network of g-C_3_N_4_ and GNF. The 3D g-C_3_N_4_/GNF/GCE was utilized for the detection of nucleic acid bases with a well resolved oxidation peak for the individual analyte. The electrocatalytic current was established to be a linear range from 0.3 × 10^–7^ to 6.6 × 10^–6^, 0.3 × 10^–7^ to 7.3 × 10^–6^, and 5.3 × 10^−6^ to 63.3 × 10^−4^ M for G, A, and T with a detection limit of 4.7, 3.5 and 55 nM, respectively. The diffusion co-efficient and the kinetic parameters were derived from the chronoamperometry technique. The proposed sensing strategy has been effectively used for the application in real sample analysis and observed that the electrode free from the surface fouling.

## Introduction

Graphitic carbon nitride (g-C_3_N_4_) is essentially linked by nitrogen atoms through a π-conjugation and covalent bonding of carbon, considered to be the most stable allotropes with chemical and high thermal stability. In addition, the tri-s-triazine units allow carbon nitride polymer formation along with adaptable electronic features^1^.Owing to the exceptional catalytic properties and inimitable optical or electronic behaviour, g-C_3_N_4_ has conquered the extensive attention in the field of catalysis, hydrogen production, sensor and degradation processes^2–5^. In contrast, the low electrical conductivity of g-C_3_N_4_ limits its application in the electrochemical sensors^6^. To address these limitations, various methods have presented in improving g-C_3_N_4_ conductivity, including metal or carbon based nanomaterials doping^7^, designing the surface heterojunctions with semiconductor^8^, copolymerization with the organic molecules^9^, and coupling with carbon materials^10^. Similar kind of carbon based graphene flakes has attracted recently in the electrochemical application^11–19^ due to the flexible structure, inherent chemical and mechanical stability, higher electronic conductivity, large surface area and the high intrinsic carrier mobility^20,21^. Herein, this study has attempted to prepare g-C_3_N_4_/GNF nanocomposites to enhance the electrical conductivity with defect-free hybrid system.

It has been demonstrated that the solvent exfoliation is considered to be best suitable method to synthesis a few-layered defect-free graphene sheet with the exceptional electronic properties. The hybrid layer-by-layer assembly of g-C_3_N_4_/GNF structure has been established by the hydrothermal method by heating of g-C_3_N_4_ and GNF facilitates the electrons movement between electrolyte and electrode interface. The present method can be applied to electrochemically detect DNA bases. The DNA bases in the form of helical structure play a vital role in all living beings for the cell signaling and cellular energy transduction^22^. It carries information concerning the inheritance, inculcates the biological production of enzymes and proteins by means of replication, transcription and genetic contents, determines the cell metabolism, and epigenetic modifications^23^. DNA shows extensive consequence on cardiac arrhythmia, cerebral and coronary circulation, and inhibition of neurotransmitter release due to the biological phenomena discrepancy in the organization of DNA, whose modifications can be interrelated with the concentration of nitrogenous bases^24^. Abnormal changes in DNA bases indicate an immune system deficiency that increase tendency towards various infectious diseases, namely AIDS, cancer, epilepsy, tumor genesis and mental retardation. Several reports revealed the quantification and detection of DNA bases using several analytical techniques, such as mass spectroscopy^25^, flow-injection chemiluminesence^26^, capillary electrophoresis^27^, liquid chromotography^28^. The above specified methods have some disadvantages of low sensitivity, complicated operating procedure, and high cost. Electrochemical method highly encouraged in DNA bases analyses and attributed for inexpensive, rapidity, least LOD, high sensitivity along with stability. The simultaneous detection of DNA bases was only accounted for by a number of electrochemical approaches^29–31^. Banks et al., have critically examined the carbon electrodes and promising the electron transfer process for sensing of DNA bases. The beneficial effect of graphene nanoflakes system towards the electrochemical recognition of DNA bases is principally owing to the large surface area as well as the edge defects are the major causes^32^.

This investigation extends this idea using the hybrid system like g-C_3_N_4_/GNF to improve the detection limit due its high surface area as well as π–π interaction within the gC_3_N_4_/GNF nanocomposites. The electrochemical oxidation of nucleic acid bases was investigated by using the electrodes modified as metal-free carbon-based hybrid nanostructures with an improved conductivity and high surface area. The electrochemical features of DNA bases in modified electrodes with nanocomposites were studied using CV and DPV techniques. An enhanced oxidation peak current of DNA bases was noted at Nafion/g-C_3_N_4_/GNF/GCE was improved greatly compared with the bare GC Electrode. The system exhibits low LOD, good stability and flexibility to evaluate A, G, and T simultaneously.

## Experimental

### Reagents

Potassium ferricyanide, graphite powder, melamine, and Whatman qualitative filter paper Grade 1 were bought from Sigma-Aldrich. Nucleic acid bases like guanine, adenine, thymine, and sodium hydroxide, potassium chloride, urea, ethanol, acetic acid, hydrochloric acid, potassium dihydrogen phosphate (KH_2_PO_4_), *N*-cetyl-*N*, *N*, *N* trimethyl ammonium bromide (CTAB), and potassium hydrogen phosphate (K_2_HPO_4_) were procured from Hi-Media, India.

### Characterizations

High resolution transmission electron microscope (HRTEM, Tecnai 30 G^2^ S-TWIN, FEI Company) and field emission scanning electron microscope (FESEM, Hitachi, Japan) analyses were performed to visualize the morphology of nanocomposites. Fourier transform infrared (FTIR, Bruker Vector-22 instrument) spectroscopy was utilized to analyze the vibrational properties between 4,000 and 400 cm^−1^. The crystallinity of samples was examined using X PERT-PRO X-ray diffractometer. The surface area was found through the adsorption data points that were obtained using BET (Brunauer–Emmett–Teller) equation at P/P_0_. X-ray photoelectron spectroscopy (XPS, Omicron Nanotechnology ESCA-14) utilized in analyzing surface compositions of samples. The software Gamry USA model 330 including PV220 software and a CHI-660B electrochemical workstation, CH instruments, Texas (USA), were used to record the electrochemical studies. BAS Pvt. Ltd., USA invented electrode systems made up of GCE with 3 mm geometric working surface was procured. GCE applied as working electrode, 0.5 mm diameter platinum as counter electrode, and Ag/AgCl with 3 M saturated KCl was utilized as reference electrode. The polishing kit purchased from bioanalytical system (BAS, USA) for polishing the GCE before the electrochemical analysis was carried out.

A buffer (neutral pH) was prepared with 0.1 M KCl, K_2_HPO_4_, and KH_2_PO_4_, using distilled deionized (DD) water in regular flask of 250 mL. The buffer pH was calculated at room temperature (RT) by means of an Elico-pH meter (Elico, Pvt. Ltd, India). Freshly prepared guanine, adenine, and thymine stock solution with 0.1 M concentration using DD water, were kept in 5 °C refrigerator.

## Electrochemical measurement parameters

The proposed modified electrode was detected using cyclic voltammetry technique (Potential window: + 0.2 to + 1.1 V, + 0.4 to 1.25 V, and 0.7 to 1.65 V for G, A, and T: Scan rate: 50 mV s^−1^), chronoamperometric curve of G, A, and T oxidation was observed at a potential step of + 0.8, + 1.0, + 1.3 V, differential pulse voltammetry (Scan rate: 20 mV s^−1^, Pulse width: 50 mV, Pulse amplitude: 25 mV) and Amperometric (i–t) curves of G, A, and T were observed at an applied potential of + 0.8, + 1.0, + 1.3 V, under hydrodynamic condition.

### Development of graphene nanoflakes (GNF)

The GNF was developed by adding 200 mg of powdered graphic in glacial acetic acid (15 mL) with 0.5 M CTAB and the compound was kept at room temperature in ultrasound bath for 3 h. The mixed composition was subsequently refluxed for a day at 100 °C under nitrogen gas. Thereafter, the solution kept at RT overnight to settle the stabilized flakes underneath of the flask. Finally, the black color GNF was centrifugally separated at 2000 rpm and then thorough cleaning with distilled water and acetone. Upon drying in vacuum oven about 80 °C, the resulting sample was isolated and stored for further investigations^19^.

### Synthesis of g-C_3_N_4_

A mixture of 0.5 g of urea and melamine was mixed in 40 mL of 80% ethanol to synthesize g-C_3_N_4_, then moved to the Teflon-lined stainless-steel autoclave for heat treatment about 6 h at 180 °C and then drying at 40 °C for 15 h using vacuum oven. The consequent solid mass was shifted in an aluminium boat and then heated in muffle furnace for further heating at 20 °C/min of heating rate with 550 °C about 3 h.

### Preparation of g-C_3_N_4_/GNF nanocomposites

An equal quantity of ethanol solution containing g-C_3_N_4_ and GNF (200 mg) was mixed and agitated under sonification for 2 h to attain an equivalent suspension, and then put in an autoclave of stainless steel lined with Teflon and heated for 5 h in a 150 °C muffle furnace. The sample was permitted to cool down to RT, followed by washing with ethanol thrice to remove unwanted particles and then allowed to dry in vacuum desiccator at RT.

### DNA sample preparation

In a real sample procedure, chicken liver and beef liver were procured from the local meat market, Chennai—600 085. About 3 gm of the meat sample was taken accurately and standardized with 15 mL buffer solution by means of mortar and pestle, which was brought to neutral pH by filtration and dilution using Whatman No.1 filter paper and buffer, respectively. In actual sample analysis 15 mL aliquot was measured and the conventional addition procedure for quantification was implemented. Finally, the chicken liver and beef liver solution were analyzed to find the DNA base levels. In the case of lower concentration ranges with nucleic acid bases below the detection limit, a standard addition method was followed.

### Development of Nafion/g-C_3_N_4_/GNF modified GCE

The Nafion/g-C_3_N_4_/GNF/GCE was invented based on the following protocol. GCE was primarily cleaned by mechanical smoothing with alumina powder paste (0.5 micron) followed by extensive rinsing with DD water subsequently cleaned with acetone and nitric acid with ratio 1:1 v/v. Finally, GCE surface was dried at RT after washing in ultrasonic DD water bath about 20 min. Around 2 mg of g-C_3_N_4_/GNF was scattered by ultrasonics in alcohol (3 mL ethanol) for 5 min. Subsequently, about 5 μL of the colloidal solution was released on the GCE working surface which was enabled to dry at RT for 30 min. After that, 1% of 3.0 μL Nafion (99.9% of 1 mL ethanol added to 0.01 mL Nafion) was dropped onto the surface of modified GCE and kept for 30 min at RT.

## Results and discussion

### Morphological studies on g-C_3_N_4_/GNF nanocomposites

**Figure 1:**
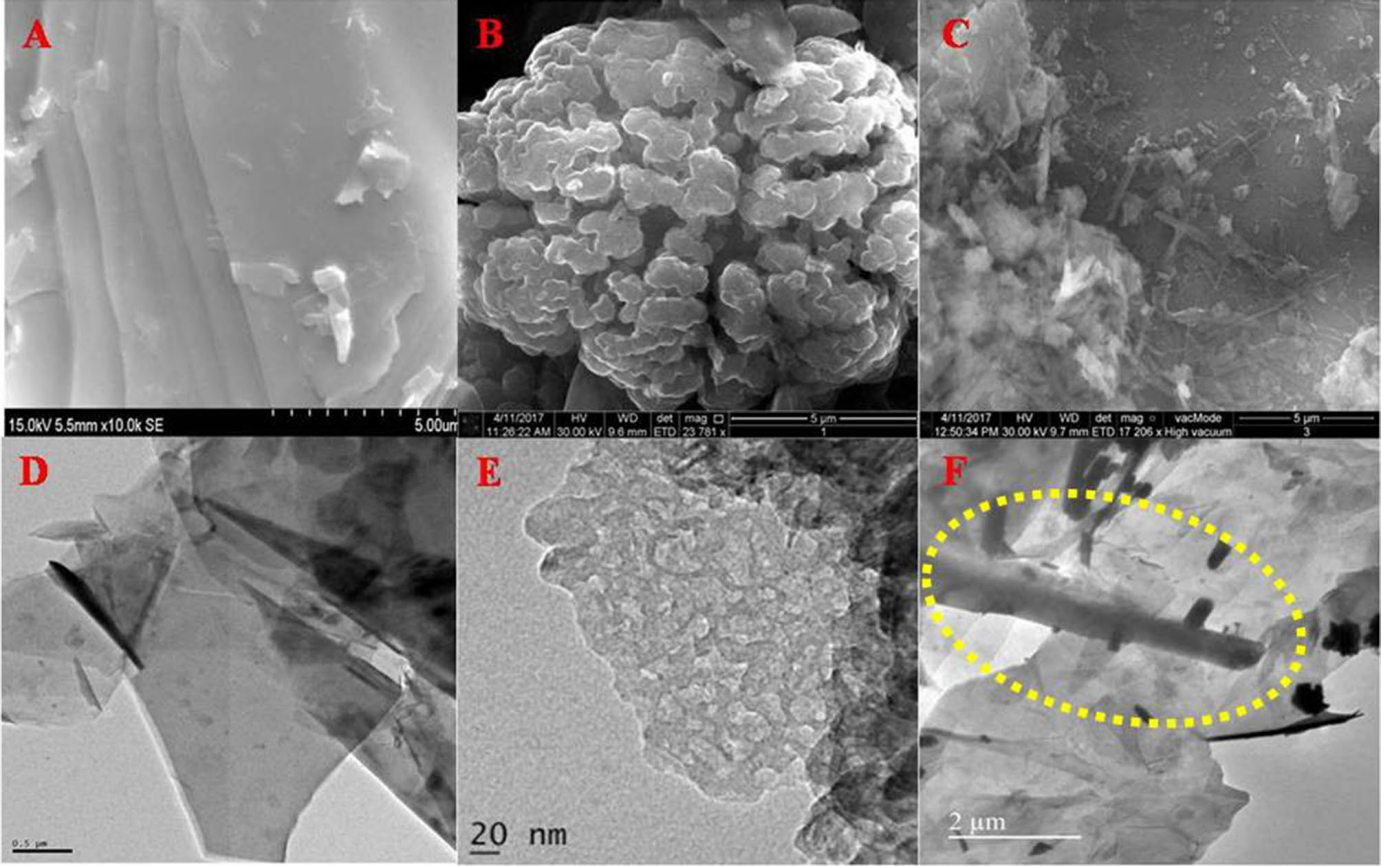
FE-SEM micrographs of (**A**) GNF, (**B**) g-C_3_N_4,_ (**C**) g-C_3_N_4_/GNF nanocomposites. HR-TEM image of (**D**) GNF, (**E**) g-C_3_N_4,_ and (**F**) g-C_3_N_4_/GNF nanostructures.

The morphological features of all the synthesized nanostructures were analysed by FE-SEM investigations using the beam of electrons as a source to scan the features present in the nanostructures. When the electron beam detaches the secondary electrons ejected from the graphitic carbon nitride, the image processed were porous sheet like architectures as depicted in Fig. 1A, while processing the secondary electrons ejected from the GNF, the morphological features were found to be bunch of GNFs (Fig. 1B). When porous g-C_3_N_4_ was incorporated into the GNF sheets in the form of hybrid nanocomposite, unique morphological features were observed as nanorods are distributed over the sheet like features of GNF sheets. During the hydrothermal treatment, the porous features of g-C_3_N_4_ interconnected with GNF and form an individual nanorod like features that are uniformly spread over the surface of GNF (Fig. 1C). Further, the nanostructures of GNF, g-C_3_N_4_ and its nanocomposite were investigated through HR-TEM micrographs. From the figures, the coherently scattered electrons present the porous sheet like features of g-C_3_N_4_ and sheet like structures of GNF, further the transparent images depicts that the sheets were very thin in thickness dimension (Fig. 1D, E). The nanocomposites are shown in Fig. 1F, in which a nanorod likes structures of g-C_3_N_4_ that were distributed on the GNF sheets (rods formation indicated in yellow circle). Further, the HR-TEM images are correlated with the FE-SEM images. These exceptional characters of the nanostructures can be used for sensing of various biomolecules.

### XRD studies

The XRD pattern was recorded to establish the lattice structure of the nanocomposites are displayed and shown in Fig. S1. The g-C_3_N_4_ showed two separate diffraction peaks which are due to the characteristic broad plane and sharp planes at 10.30° and 27.10° respectively. The low-angle Bragg peak located at 10.30° was recognized as (100) plane with the subsequent inter-planar spacing of *d* = 7.79 nm ensuing from the trigonal nitrogen linkage of tri-s-triazine units. A sharp Bragg peak at 27.10° correspond to *d* = 7.98 nm attributed to a long-range inter-planar stacking plane in conjugated aromatic systems that was recognized as (002) plane^33,34^. GNF showed a little intensity broad plane and the shifting in (002) plane. Moreover, in the XRD pattern, shifting towards a lower angle side was observed in Bragg peaks. This wider peak is mainly due to the firm GNF structure^35^. However, the g-C_3_N_4_/GNF nanocomposites with a sharp peak at 26.63° that evidently represent g-C_3_N_4_ and GNF form of the nanocomposites through chemical bonds.

### FT-IR spectral studies

FT-IR studies were implied to analyze the functional bonds existing in g-C_3_N_4_, GNF and g-C_3_N_4_/GNF nanocomposites that were shown in Fig. S2. In g-C_3_N_4,_ widening peaks from 3,010 to 3,414 cm^−1^resemble the expansion of N–H and O–H due to physical absorption of water molecules^36,37^. A peak at 800 cm^−1^ indicates signal of heterocycles existing in the g-C_3_N_4_ that are reflecting to the breathing modes of s-triazine unit as well as *sp*^2^ –C=N–. The weak peaks located at 1,081, 1,221 and 1,319 cm^−1^ attribute C–N heterocycle’s stretching. The peaks situated at 1,367, 1,382, 1,420 cm^−1^ specify the amorphous *sp*^3^ C–C bond^38,39^. In GNF, exhibiting absorption peaks represent C=O and C–O groups that were appeared at 1,331 cm^−1^. The 112, 855 and 673 cm^−1^ bands confirmed the occurrence of epoxy that complies with the vibrations of symmetric stretching, asymmetric stretching and deformation, respectively. In addition, the broad characteristic band of O–H stretching and the weakly engaged water molecules exhibit at 3,432 cm^−1^. Furthermore, by comparing g-C_3_N_4_ with g-C_3_N_4_/GNF nanocomposites additional dual new peaks are found at 800 and 1,319 cm^−1^, that reflect characteristic of s-triazine units and –C–N heterocycles stretching vibration.

### BET surface area measurements

**Figure 2:**
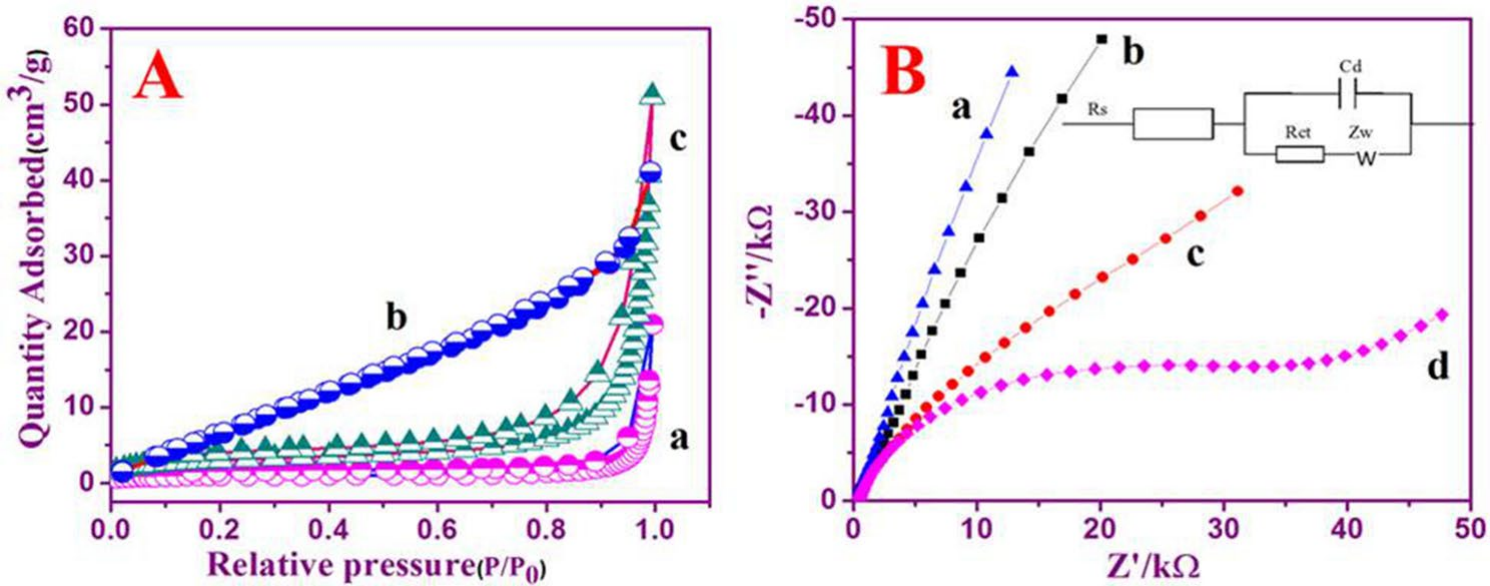
N_2_ adsorption–desorption of isothermal curves of (a) g-C_3_N_4_, (b) GNF, and (c) g-C_3_N_4_/GNF nanosheets. Nyquist plots of, (a) bare GCE, (b) GNF/GCE, (c) g-C_3_N_4_/GCE and (d) Nafion/g-C_3_N_4_/GNF/GCE in 10 mM [Fe(CN)_6_]^3-/4-^ along with 0.1 M of KCl as the redox probe. AC amplitude is 5 mV; frequency range between 0.01 Hz and 100 kHz; figure inset shows Randles equivalent circuit.

BET employed in evaluating the working area of the g-C_3_N_4_, GNF and g-C_3_N_4_/GNF. The nitrogen adsorption and desorption isotherms of (a) g-C_3_N_4_, (b) GNF, and (c) g-C_3_N_4_/GNF represented in Fig. 2A, B. In accordance to IUPAC classification, all samples obey the type—IV, N_2_ adsorption–desorption isotherm accompanied by a type H3 hysteresis loop that is mainly accredited to the predominance of mesoporous. The surface area values were measured from the isotherms of g-C_3_N_4_, GNF and g-C_3_N_4_/GNF nanocomposites that were found as 3.5, 36.2 and 11.25 m^2^ g^−1^, respectively. The GNF’s surface area is found to be higher than the nanocomposites sample, because g-C_3_N_4_ surface area is comparatively very small, so the addition of GNF into g-C_3_N_4_ nanostructure has further enhanced g-C_3_N_4_'s surface area.

### X-ray photoelectron spectroscopy studies

**Figure 3:**
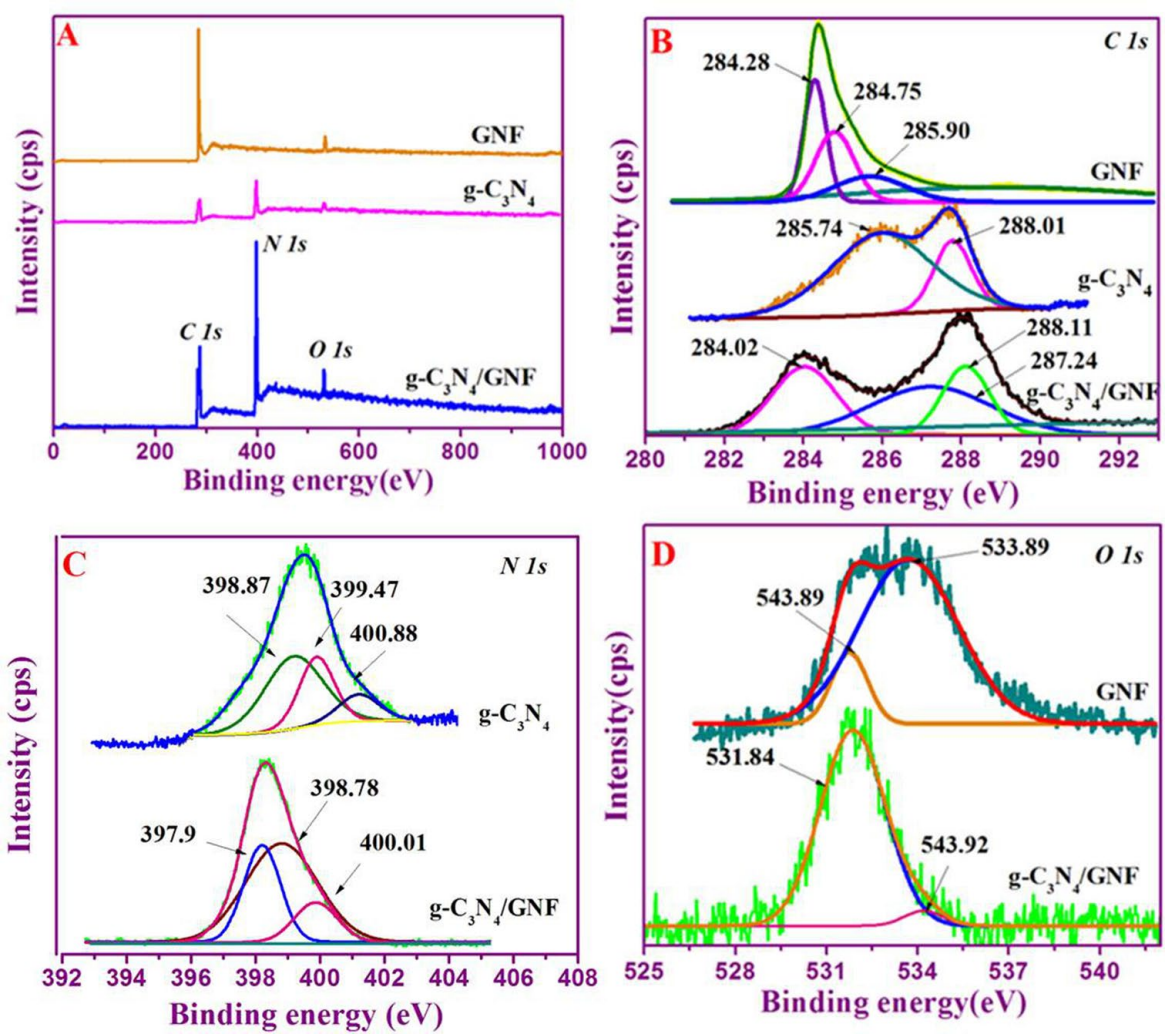
XPS survey spectra of GNF, g-C_3_N_4_, g-C_3_N_4_/GNF (**A**), High- resolution C 1*s* of GNF, g-C_3_N_4_, g-C_3_N_4_/GNF (**B**), High-resolution N 1*s* of GNF, g-C_3_N_4_, g-C_3_N_4_/GNF (**C**), and High- resolution O 1*s* of GNF, g-C_3_N_4_, g-C_3_N_4_/GNF (**D**).

XPS analyses the surface configuration in g-C_3_N_4_, GNF, g-C_3_N_4_/GNF nanostructures and the functional bonding occur between the elements in the nanocomposites, and their findings are shown in Fig. 3A–D. Survey scan spectra of GNF exhibited the presence of C and O elements, while the spectra of g-C_3_N_4_ shown N, C and O existences. In g-C_3_N_4_/GNF nanocomposites, XPS analysis was performed, in which the composition is including C, O and N in the nanocomposite. Further, the high-resolution scans were performed (Fig. 3A). The deconvoluted C 1*s* peak revealed two distinctive peaks at 285.74 and 288.01 eV for g-C_3_N_4_, conform the graphitic carbon as well as *sp*^2^ bonded carbon in its heterocycle frameworks (N–C=N), accordingly^40^. These features are similarly noticed in C 1*s* peak of g-C_3_N_4_/GNF nanocomposites (Fig. 3B). Conversely, the high-resolution C 1*s* peak of GNF indicated three distinctive peaks present at binding energies of 284.28, 284.75 and 285.90 eV, which coincide graphitic carbon, C–OH and C=O groups, respectively. To evaluate the character of nitrogen occurred in the samples, high resolution scan was carried out for N 1*s*, g-C_3_N_4_ displayed three unusual nitrogen centers positioned at 398.78, 400.1, and 397.9 eV that corresponds to pyrrolic, pyridinic nitrogen, graphitic and amino respectively^41,42^. N 1*s* spectra of the nanocomposite displayed a peak centered at 398.78 eV that corresponds to the intermolecular interaction between graphitic carbon and nitrogen species of two nanostructures (Fig. 3C). High-resolution O 1*s* of GNF, g-C_3_N_4_, g-C_3_N_4_/GNF is shown in Fig. 3D. It has already been established that the occurrence of a variety of nitrogen centers will deviate the state density and hurry the graphic carbon electronic cloud thus improving conductivity and electrochemical properties of g-C_3_N_4_^43^. In summary, the average chemical composition and their corresponding bonding nature can be derived from the XPS analysis.

### EIS studies

EIS investigates the coherent phenomena of all modified electrodes. To investigate nanocomposites Nafion/g-C_3_N_4_/GNF/GCE, the impedance spectrum was examined to detect differences in the electrode through the amendment steps. Nyquist plot displays the model circuit along the charge transfer resistance (R_ct_), the double layer capacitance (C_d_), the Warburg impedance (Z_w_), and the solution resistance (R_s_), which mirror electron shifting characteristics based on actual [Fe(CN)_6_]^3-/4-^redox probe. The parameters used were 5 mV AC signal amplitude in the 0.01–100 Hz frequency range, with [Fe(CN)_6_]^3-/4-^ sustaining KNO_3_ redox probe. The electron transfer resistance of bare GCE, GNF/GCE, g-C_3_N_4_/GNF and Nafion/g-C_3_N_4_/GNF/GCE obtained were 5 kΩ, 9 kΩ, 12 kΩ, and 40 kΩ, respectively (Fig. 2B). The EIS response for bare electrode, GNF/GCE and g-C_3_N_4_/GCE exhibited nearly a straight line, indicating almost non-heterogeneous charge transfer resistance. Semicircular portions of the modified GCE suggesting the transfer of electron limited process and the interfacial resistance of Nafion/g-C_3_N_4_/GNF/GCE was greater compared to other modified electrodes as the resistance is applied to the electrode, resulting in a reduced rate of electron movement. This results show the redox probe [Fe(CN)_6_]^3-/4-^ exhibit a rapid charge transfer kinetics at Nafion/g-C_3_N_4_/GNF/GCE.

### Electrochemical characteristics of modified electrodes

The electrochemical characteristics of GC electrodes with different modification were examined by CV using [Fe(CN)_6_]^3-/4^ in KCl as redox probe with a scan rate of 50 mV s^−1^. A bare GCE has reversible redox features that was sensible for [Fe(CN)_6_]^3-/4^ probe with a peak separation ($\Delta$E_p_) at 80 mV [Fig. S3A(a)] while GNF/GCE shows reversible redox peaks and peak separation at 90 mV [Fig. S3A(b)]. Figure S3A(c) represents g-C_3_N_4_/GCE together with the reversible process exhibit a peak separation at 100 mV. Besides, Nafion/g-C_3_N_4_/GNF/GCE exhibits a well pair of redox peaks through a cathodic and anodic contributions at + 0.083 and + 0.153 V (vs. Ag/AgCl), respectively. Potential peak separation value of anodic and cathodic peak potentials (E_p_ = E_pa _− E_pc_) at 50 mV s^−1^ is close to 70 mV. In addition, the redox peak maximum current ratio ((I_pa_/I_pc_ ≈ 1) describes the reversible electrochemical activity with one electron transfer cycle [Fig. S3A(d)]. From CV curves it can be observed the bare GCE, GNF/GCE, g-C_3_N_4_/GCE exhibits an extremely low background current whilst Nafion/g-C_3_N_4_/GNF/GCE displays a much higher background peak current with less peak separation value.

The effective working area of different modified electrodes has been computed by Randles–Sevick equation given in the following relation^44^.

1 $$ {\text{I}}_{{\text{p}}} = \left( {2.69 \times 10^{5} } \right){\text{n}}^{3/2} {\text{AD}}_{{\text{o}}}^{1/2} {\text{C}}_{{\text{o}}}\upupsilon ^{1/2}$$

where ‘I_p_’ stands for ferricyanide redox peak current, ‘n’ stands for total electrons moved within the redox cycle, ‘C_o_’ means concentration redox probe’s (mol cm^−3^), ‘D’ means diffusion coefficient (6.70 ± 0.02) × 10^–6^ cm^2^ s^−1^) of ferricyanide, and υ stands for scanning rate (mV s^−1^). ‘A’ is the electrode’s actual working area to be calculated. Electroactive working area of Nafion/g-C_3_N_4_/GNF/GCE was determined to be 3.2 × 10^–4^ cm^2^ in ferricyanide solution. The effective electroactive surface area signifies the superior electrochemical reactivity of Nafion/g-C_3_N_4_/GNF/GCE.

The electrochemical properties and reversibility of Nafion/g-C_3_N_4_/GNF/GCE were performed by continuous scanning of the electrode about 50 cycles at 50 mV s^−1^ scanning rate. Figure S3B represents the redox activity of the redox probe on Nafion/g-C_3_N_4_/GNF/GCE indicates the excellent electrochemical stability. The redox probe and the impact of the scanning rate on the peak signal were investigated by the variation in scanning rate at Nafion/g-C_3_N_4_/GNF/GCE to analyze the characteristic of the modified GCE electron transfer cycle. The redox peak signal rises and the peak potentials of anodic and cathodic contributions (E_pa_ and E_pc_) were also increases. In addition, the peak separation was observed at all scan rates varying between 10 and 400 mV s^−1^ (Fig. S3C). The peak current (I_pa_ and I_pc_) indicated a linear relation with the square root of the scan rate and a linear regression equation of I_pa_ (μA) = 4.8464 (υ^1/2^/mV^1/2^ s^−1/2^) − 11.3151 and I_pc_ = (μA) = − 3.9703 (υ^1/2^/mV^1/2^.s^−1/2^) + 5.9466 with a regression coefficient of R^2^ = 0.9914 and 0.9934 (Inset: Fig. S3C). These findings clearly show that the redox reaction is a diffusion controlled. Furthermore, a log current versus log υ indicates a linear scheme with corresponding linear regression equation of log I_p_/μA = 0.5847 log υ + 0.5493 with regression coefficient of R^2^ = 0.9993 (Fig. S3D).

### Electrochemical oxidation of DNA bases at Nafion/g-C_3_N_4_/GNF/GCE

Electrocatalytic oxidation of DNA base molecules at Nafion/g-C_3_N_4_/GNF modified GCE was investigated for the oxidation peak current value of the DNA base ‘G’ (guanine). Figure S4A, represents the cyclic voltammetry response of ‘G’ on (a) bare GCE, (b) bare GCE in the existence of 3.3 × 10^–4^ M of guanine and, (c) Nafion/g-C_3_N_4_/GNF/GCE in the existence of 3.3 × 10^–4^ M of ‘G’ in 0.1 M buffer (neutral pH) at 50 mV s^−1^. Bare GCE evinces less intensity anodic oxidation peak current value along the peak potential at + 0.79 V against Ag/AgCl electrode, revealing the insignificant electrocatalytic characteristics towards ‘G’ oxidation. The Nafion/g-C_3_N_4_/GNF/GCE electrode indicates a rapid current signal with an oxidative peak potential at + 0.8 V against Ag/AgCl and the oxidation signal is three times higher than bare GCE’. The oxidation peak signal rises with constant elevation in ‘G’ concentration and a linear relationship from 1 × 10^–4^ to 14 × 10^–3^ M (Fig. S4B). The linear plot of I_pa_ versus conc. of ‘G’ (Inset: Fig. S4B).

A similar tendency was noticed in the electrochemical oxidation of ‘A’ (adenine) using Nafion/g-C_3_N_4_/GNF/GCE. Electrochemical characteristics of ‘A’ on, (a) bare GCE, (b) bare GCE in the existence of 3.3 × 10^–4^ M of ‘A’, and (c) Nafion/g-C_3_N_4_/GNF/GCE in the existence of 3.3 × 10^–4^ M of ‘A’ in 0.1 buffer(neutral pH), at 50 mV s^−1^ (Fig. S4C). In bare electrode, ‘A’ shows a small oxidation peak current value along with the peak potential at + 0.95 V against Ag/AgCl. Moreover, the modified Nafion/g-C_3_N_4_/GNF/GCE in the existence of ‘A’ displays a sharp oxidation peak response along with peak potential at + 1.0 V against Ag/AgCl electrode. Additionally, linear peak current values were observed, increasing in ‘A’ concentration while increasing the concentration ranges from 6.0 × 10^–7^ to 20 × 10^–5^ M along with elevation of oxidation peak current (Fig. S4D) and the corresponding linear plot of I_pa_ versus conc. of ‘A’ is shown in Fig. S4D.

### Influence of scan rate

**Figure 4:**
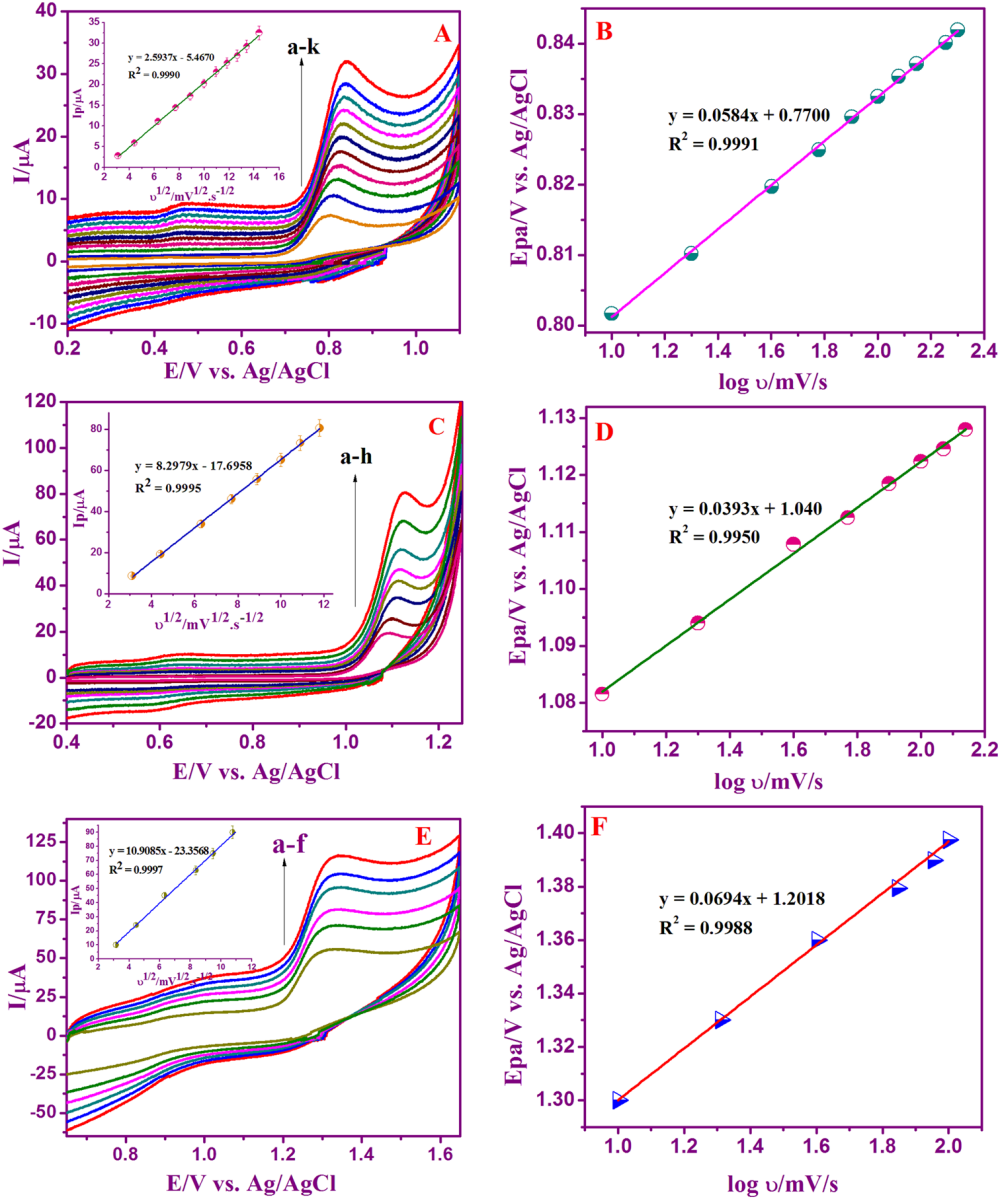
Cyclic voltammograms of Nafion/g-C_3_N_4_/GNF/GCE at various scan rates (10–240 mV s^−1^) with 3.3 × 10^–4^ M of ‘G’ (**A**), (10–140 mV s^−1^) with 3.3 × 10^–4^ M of ‘A’ (**C**), and (10–120 mV s^−1^) with 6.6 × 10^–4^ M of ‘T’ (**E**) in 0.1 M buffer (neutral pH). Linear plot E_pa_ versus log υ (**B**, **D**, **F**). Insets: Linear plot showing I_pa_ versus υ^1/2^.

The consequence of oxidation peak current responses of G, A, and T at Nafion/g-C_3_N_4_/GNF modified GCE at different scan rate was recorded by cyclic voltammetry. To acquire the kinetic parameters, CV curves of Nafion/g-C_3_N_4_/GNF/GCE in the 0.1 M buffer (neutral pH) along with 3.3 × 10^–4^ M of ‘G’ and A, 6.6 × 10^–6^ M of ‘T’ were investigated at different scan rates. CV characteristics of G, A, and T substantiate that merely oxidation peaks were noticed representing irreversible electrochemical oxidation of A, T and G. Figure 4A, C, and E exhibit that with variation in scanning rate, inflation of the oxidation peak response observed. The anodic peak currents of A, G and T found to be linearly related to the scan rate. The linear plot of I_p_ versus υ^1/2^, displays an outstanding linear relationship (Figure insets in 4A, C, E) showing that electrochemical oxidation of G, A, and T is a process purely controlled by adsorption^45^. The linear regression equation is as follows:

$$ \begin{aligned} {\text{I}}_{{\text{p}}} /\upmu {\text{A}}\;\left( {\text{G}} \right) & = 2.5937\upupsilon ^{1/2} - 5.4670\quad (R^{2} = \, 0.9990) \\ {\text{I}}_{{\text{p}}} /\upmu {\text{A}}\;\left( {\text{A}} \right) & = 8.2979\upupsilon ^{1/2} - 17.6958\quad (R^{2} = \, 0.9995) \\ {\text{I}}_{{\text{p}}} /\upmu {\text{A}}\;\left( {\text{T}} \right) & = 10.9085\upupsilon ^{1/2} - 23.3568 \quad (R^{2} = \, 0.9997) \\ \end{aligned}$$

In addition, Fig. 4B, D, and F also confirms the oxidation peak potential of DNA bases against scan rate. An excellent relation was obtained with the E_pa_ versus log scan rate associated with linear regression equation as:

$$ \begin{aligned} {\text{I}}_{{\text{p}}} /\upmu {\text{A}}\;\left( {\text{G}} \right) & = 0.0{\text{584 log}}\upupsilon \, + \, 0.{77}00\quad ({\text{R}}^{{2}} = \, 0.{9991}) \\ {\text{I}}_{{\text{p}}} /\upmu {\text{A}}\;\left( {\text{A}} \right) & = 0.0{\text{393 log}}\upupsilon \, + { 1}.0{4}00\quad ({\text{R}}^{{2}} = \, 0.{995}0) \\ {\text{I}}_{{\text{p}}} /\upmu {\text{A}}\;\left( {\text{T}} \right) & = 0.0{\text{694 log}}\upupsilon \, - { 1}.{2}0{18}\quad ({\text{R}}^{{2}} = \, 0.{9988}) \\ \end{aligned} $$

By investigating the interrelation between the peak current and its oxidation potential, the electrochemical parameters are related to the rate constant of standard electrode reaction (k_s_), charge transfer coefficient (α), electron shifting related in the rate determination step (n) and concentration of surface adsorbed (Γ) can be computed, at its scan rate by using Laviron’s equation^46,47^.

2 $$ {\text{E}}_{{\text{p}}} = {\text{E}}^{{\text{o}}} + ({2}.{3}0{\text{3RT}}/\upalpha {\text{nF}}){\log}({\text{RTk}}_{{\text{s}}} /\upalpha {\text{nF}}){-}({2}.{3}0{\text{3RT}}/\upalpha {\text{nF}}){\log}\upupsilon  $$

3 $$ {\text{I}}_{{\text{p}}} = {\text{n}}^{{2}} {\text{F}}^{{2}} {\text{A}}\Gamma\upupsilon /{\text{4RT}} $$

4 $$ {\text{Log k}}_{{\text{s}}} = \upalpha {\log}({1} - \upalpha ) + ({1} - \upalpha ){\log}\upalpha  {-}{\log}({\text{RT}}/{\text{nF}}\upupsilon ){-}\upalpha ({1} - \upalpha ){\text{nFE}}_{{\text{p}}} /{2}.{3}0{\text{3RT}}$$

where υ is the scan rate and E^o^ stands for formal potential of peaks. F, R and T are the standard constants with value of 96,500 C mol^−1^, 8.314 J mol^−1^ K^−1^, and 273 K, respectively. Usually, for irreversible electrode activity, α is presumed to be 0.5. The value of ‘n’ was computed from of the slope of E_p_ versus log υ. α was determined to be 0.48, 0.40, and 0.59 for G, A and T, accordingly. Moreover, the number of electron transferred was determined to be 2.07, 3.70, and 1.41 for A, G and T respectively, indicating that two-electron transfer represents the rate-determining step throughout the electrochemical oxidation of G, A, and T. The adsorption amount of G, A, and T on the Nafion/g-C_3_N_4_/GNF altered electrode was determined using Eq. (3). With respect to the I_p_ *vs* scan rate, the concentration of surface adsorbed (Γ) of G, A, and T was calculated as 5.14 × 10^–7^, 3.44 × 10^–6^, and 1.52 × 10^–6^ mol cm^−2^, accordingly. The rate constant standard electrode reaction (k_s_) for G, A, and T in relation to the Laviron Eq. (4), was determined to be 0.569, 1.22, and 0.730 s^−1^ respectively.

### Impact of pH on electrochemical oxidation of nucleic bases

**Figure 5:**
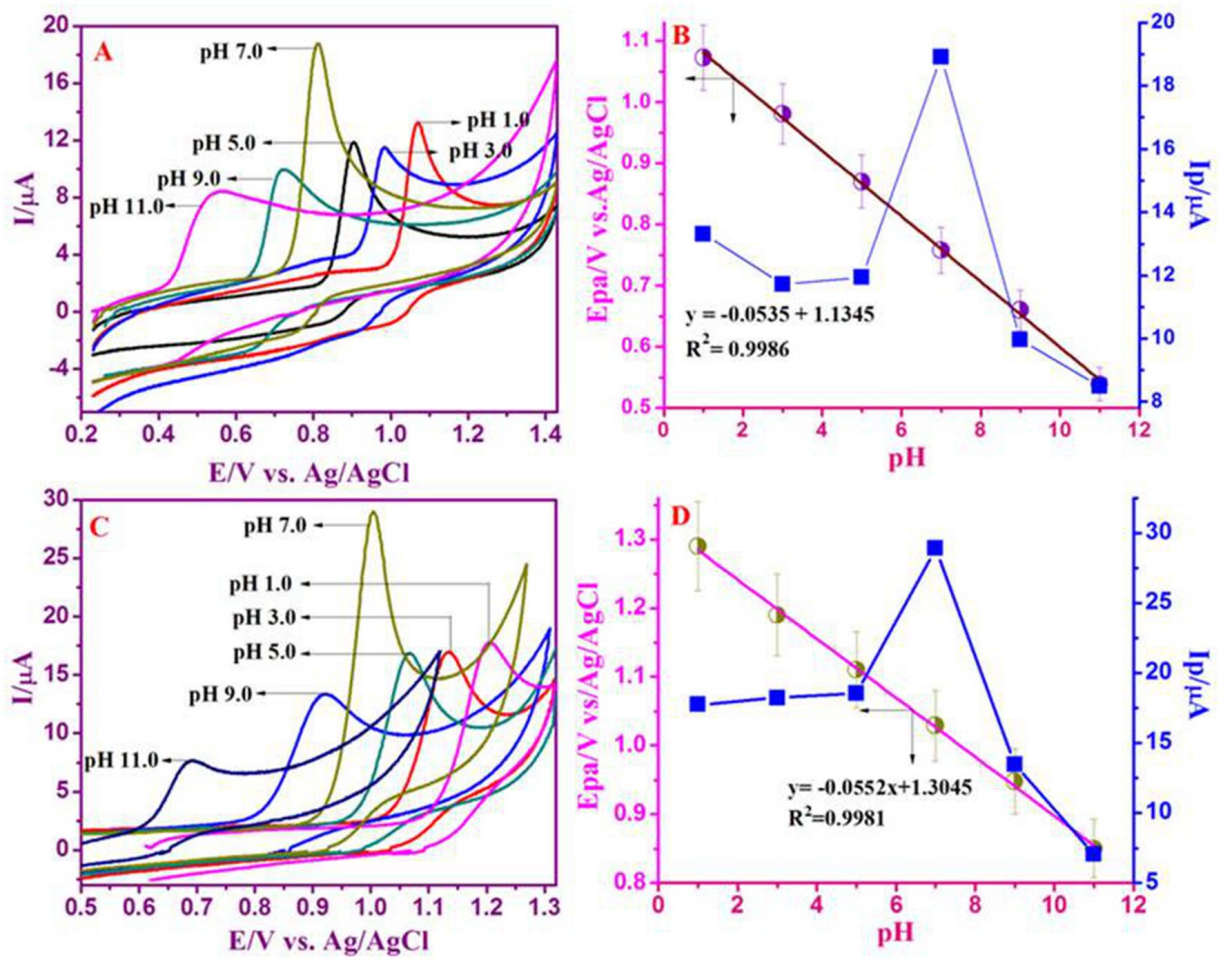
Effect of pH on CV of 3.3 × 10^–4^ M of ‘G’ and ‘A’ at Nafion/g-C_3_N_4_/GNF/GCE in the existence of 0.1 M buffer with different pH values (pH 1.0 to 11.0) at the scan rate of 50 mV s^−1^(**A**, **C**). Relationship of I_pa_ versus pH and Plot of E/V versus pH (**B**, **D**) are shown.

The consequence of pH is taking place in the electrochemical behavior of the Nafion/g-C_3_N_4_/GNF/GCE with 3.3 × 10^–4^ M of A and G was also measured. The oxidation peak current of G and A was tested at variant pH values, ranging from 1 to 11 (Fig. 5A, C). The oxidation peak potential (E_pa_) of G and A with decreasing of pH get moved positively, representing that the electrochemically oxidized G and A in reaction with the proton-transfer process. The corresponding calibration plot of E_pa_ versus pH and I_pa_ versus pH are represented in Fig. 5B, and D. The regression equation is exhibited as: E_pa_, Guanine = − 0.0535 pH + 1.1345, (R^2^ = 0.9986); E_pa_, Adenine = − 0.0552 pH + 1.3045 (R^2^ = 0.9981).

A slope value of 53 mV pH^−1^ for ‘G’ is close to the predicted with theoretical Nernstian value of 59 mV pH^−1^ (27 °C), specifying an equivalent number of electrons and proton movement. The electrochemical detection of ‘G’ shadowed two-step mechanism concerning the whole reduction of 4e^−^ as well as initial 2e^−^ oxidation as a rate determining step^48,49^. With that, deviation of 53 mV pH^−1^ demonstrated that two protons are taking action that was in excellent correlation with the oxidation system of ‘G’, as reported^49^. Similar results were obtained for ‘A’ under the identical experiential. Computed slope value (55 mV pH^−1^) is analogous to the Nernstian theoretical estimation (59 mV pH^−1^). According to Nernstian equation, the direct electro-oxidation of ‘A’ is 2 protons and electrons involved. The electrochemical mechanism of ‘A’ entails an overall six protons and electrons in the three-step operation^50,51^. It should be observed that from pH 1 to 11, the oxidation peaks DNA bases (G and A) were examined. To simulate the physiological environment, neutral pH was used as supporting electrolyte during the experiment owning to our preference for the wider separation and the highest peak currents.

### Chronoamperometry studies

**Figure 6:**
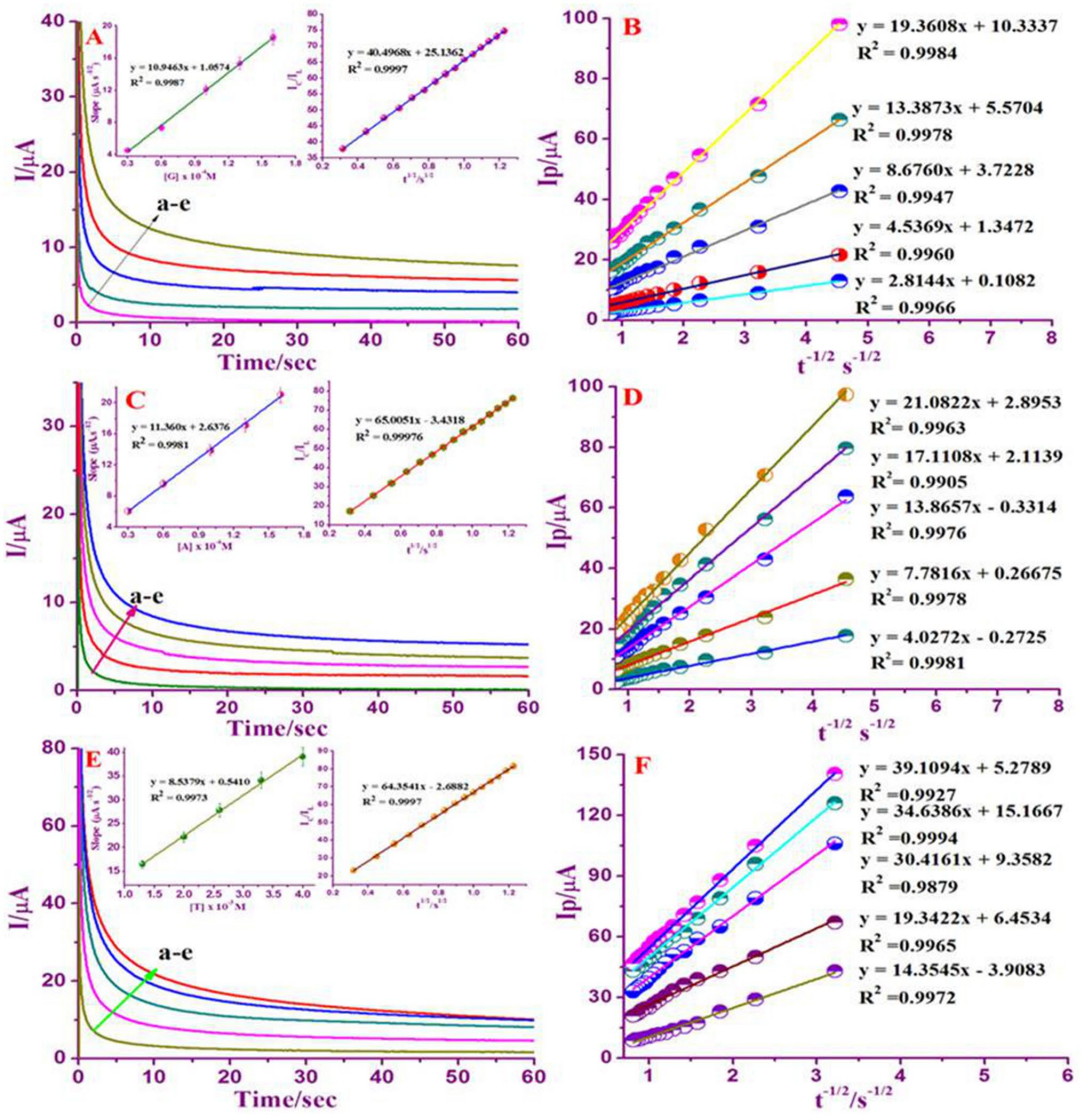
Chronoamperograms behavior of Nafion/g-C_3_N_4_/GNF/GCE in the existence of 0.3 × 10^–4^ to 1.6 × 10^–4^ M for ‘G’ and ‘A’, and 1.3 × 10^–3^ to 4.0 × 10^–3^ M for ‘T’ in 0.1 M buffer (neutral pH) at potential step of + 0.8 V, + 1.00 V, and + 1.3 V against Ag/AgCl (**A**, **C**, **E**). Inset shows plot of the linear segments slopes versus G, A, and T concentrations and outline of I_C_/I_L_ versus t^1/2^. Cottrell plots drawn with data’s were obtained from chronoamperograms (a–e) of chronoamperograms (**B**, **D**, **F**).

The catalytic oxidation of G, A, and T at Nafion/g-C_3_N_4_/GNF/GCE was investigated through chronoamperometry technique. The chronoamperograms were attained at electrode potential step from 0.0 to + 0.8 V, + 1.0 and to + 1.3 V against Ag/AgCl at various molarity of 0.3 × 10^–4^ to 1.6 × 10^−4^ M for ‘G’ and ‘A’, and 1.3 × 10^–3^ to 4.0 × 10^−3^ M for ‘T’ with existence of 0.1 M neutral buffer (Fig. 6A, C, E). In choronoamperometric studies, the diffusion coefficient (D) of A, G and T were calculated using I versus t^−1/2^ plot generated by analyzing graph from (a) to (e) which resulted in the linear lines (Fig. 6B, D, F). Plots are showing the linear graphs with a slope of the linear segments versus concentrations of G, A, and T as displayed in (Inset Fig. 6A, C, E).

As per Cottrell equation^52^,

5 $$ {\text{I}} = {\text{n}}\;{\text{F}}\;{\text{A}}\;{\text{C}}\;{\text{D}}^{{{1}/{2}}} /(\uppi ^{{{1}.{2}}} \,{\text{t}}^{{{1}/{2}}} )$$

where ‘F’ as Faraday constant (C mol^−1^), ‘A’ as electrode’s area in (cm^2^), ‘n’ as number of electrons transferred in DNA bases oxidation, ‘C’ as mass concentration of DNA bases (mol cm^−3^) and finally ‘D’ represents the diffusion coefficient (cm^2^ s^−1^). The diffusion coefficient (D) value can be obtained from the slopes of the linear plot (I vs. t^−1/2^) for G, A and T. The mean (D) value of G, A, and T was measured as 11.3 × 10^–6^, 12.2 × 10^–6^ and 6.9 × 10^–6^ cm^2^ s^−1^ corresponding to Nafion/g-C_3_N_4_/GNF/GCE.

Moreover, chronoamperometry technique was practiced to calculate the rate constant of DNA bases at Nafion/g-C_3_N_4_/GNF/GCE (inset: Fig. 6A, C, E). The rate constant of catalytic reaction was calculated by the method described below^53^

6 $$ {\text{I}}_{{\text{C}}} /{\text{I}}_{{\text{L}}} =\upgamma ^{{{1}/{2}}} [\uppi ^{{{1}/{2}}} {\text{erf }}(\upgamma ^{{{1}/{2}}} ) + {\exp}( -\upgamma /\upgamma ^{{{1}/{2}}} ) $$

where I_C_ as catalytic current and I_L_ as limiting currents of Nafion/g-C_3_N_4_/GNF/GCE in the existence and non-existence of A, G, and T is γ = k_h_C_b_t (C_b_ is the analyte molarity) is the contention that an error function is almost equivalent to one and that equation above written as:

7 $$ {\text{I}}_{{\text{C}}} /{\text{I}}_{{\text{L}}} =\upgamma ^{{{1}/{2}}}\uppi ^{{{1}/{2}}} = (\uppi {\text{k}}_{{\text{h}}} {\text{C}}_{{\text{b}}} {\text{t}})^{{{1}/{2}}}$$

where t and k_h_ are represent the time elapsed (s) and catalytic rate constant (cm^3^/mol/s) respectively. From the above equation it is implied that to find out the rate constant occurring in catalytic mechanisms (k_*h*_), where k_*h*_ of G, A and T were computed as 2.5 × 10^6^, 4.1 × 10^6^, and 4.0 × 10^6^ M^−1^ s^−1^ at Nafion/g-C_3_N_4_/GNF/GCE.

### Parallel detection of A, G and T

**Figure 7:**
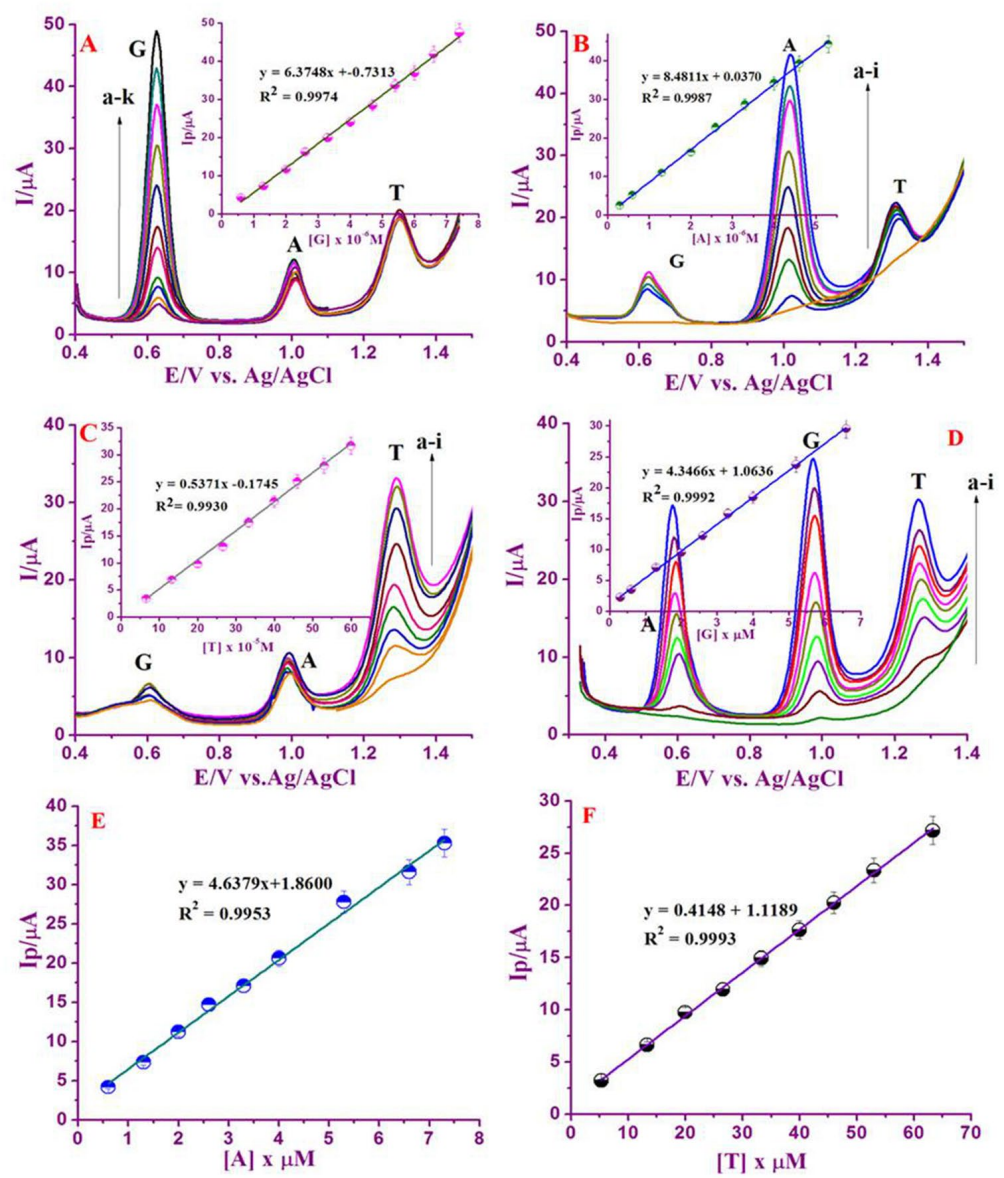
DPV at Nafion/g-C_3_N_4_/GNF/GCE in the existence of 0.1 M buffer with neutral pH, a) by changing the concentrations of ‘G’ in the existence of 50 μL of ‘A’, and 100 μL ‘T’ (**A**). Inset: Linear plot of I_pa_ versus Conc. of ‘G’, by changing the concentrations of ‘A’ in the existence of 50 μL ‘G’, 100 μL ‘T’ (**B**). Inset: Plot of I_pa_ versus Conc. of ‘A’, by changing the concentration of ‘T’ in the existence of 50 μL of ‘A’ and ‘G’ (**D**). Inset: Plot of I_pa_ versus conc. of ‘T’. DPVs of the ternary mixtures (having different concentrations) of G, A and T at Nafion/g-C_3_N_4_/GNF modified GCE (**D**).Inset: Plot of I_pa_ versus conc. of ‘G’. Plot of I_pa_ versus conc. of ‘A’ (**E**). Plot of I_pa_ versus conc. of ‘T’ (**F**). DPV parameters chosen as pulse amplitude; 25 mV, pulse width: 50 mV, scan rate: 20 mV s^−1^.

At the optimum experimental condition, the electrochemical behavior of Nafion/g-C_3_N_4_/GNF/GCE in neutral buffer (0.1 M) ternary mixtures was studied. The concentration of the selected base was assorted, but concentrations of remaining two DNA bases were reserved as constant (Fig. 7A–C). Well-defined peak potentials at + 0.60, + 1.0 and + 1.3 V were examined that corroborates to DNA bases oxidation. Meanwhile, when ‘G’ concentration was raised linearly, the oxidation peak current response in correspondence to ‘G’ was raised but the current response in correspondence to the remaining two DNA bases were nearly identical. The corresponding calibration graph of I_p_ versus conc. of ‘G’, related measurements was also executed for the individual verification of A and T at Nafion/g-C_3_N_4_/GNF/GCE (Inset Fig. 7A–C). The analytical constraints including, linear range (μM), regression coefficient (R^2^), linear regression equation, limit of detections (LODs) are tabulated (Table S1) in contrast to the LOD output of numerous modified electrodes as previously are displayed in Table 1. Comparatively, our system exhibited relatively excellent sensitivity, low limit of detection and wide range of concentration.

**Table 1:** Presenting a comparison of different chemically modified electrodes used for both individual as well as simultaneous detection of DNA bases.

^a^Electrode	Method	DNA bases	Ep(V)	Linear range μM		Limit of detection (µM)		Sample	R^2^	Ref
				Individual	Simultaneous	Individual	simultaneous			
GCE/GMC^@^	DPV	G	0.65	25–200 μM	–	–	–	Calf thymus DNA	0.998	^54^
		A	0.85	25–150 μM	–	–	–		0.987	
		T	1.12	0.2–1.4 mM	–	–	–		0.994	
Nano-ZnS/PEDOT/rGO/GCE^@^	DPV	G	0.72	0.5–150	–	0.116	–	Herring sperm	–	^28^
		A	1.03	0.5–150	–	0.141	–		–	
		T	1.20	5.0–600	–	0.257	–		–	
PSSA-ss DNA/GCE*	DPV	G	0.693	4.0 × 10^–8^–1.1 × 10^–6^	6.5 × 10^–8^–1.1 × 10^–6^	1.3 × 10^–8^	2.2 × 10^–8^	–	0.997	^29^
		A	0.982	6.5 × 10^–8^–1.1 × 10^–6^	6.5 × 10^–8^–1.1 × 10^–6^	2.2 × 10^–8^	2.2 × 10^–8^		0.997	
		T	1.244	4.1 × 10^–6^–2.7 × 10^–5^	4.1 × 10^–6^–2.7 × 10^–6^	1.3 × 10^–6^	1.4 × 10^–6^		0.995	
TiO_2_NPs-MgY/ZM CPE*	DPV	G	0.66	0.1–10	0.1–7	0.013	0.012	Ntrk2 gene of mouse	0.995	^30^
		A	0.91	0.1–10	1–6	0.02	0.132		0.991	
		T	1.12	8–10,009–600		0.878	0.878		0.998	
MWCNTs-PNF/GCE^@^	DPV	G	0.66	0.1–8.5	–	97.92	–	ssDNA	0.515	^55^
		A	0.98	0.01–3.8	–	8.7	–		0.987	
		T	0.11	0.02–7.7	–	18.7	–		0.946	
^@^Gr/IL/CHIT/GCE	DPV	G	0.74	2.5–150	–	0.75	–	Fish DNA	–	^56^
		A	1.04	1.5–350	–	.45	–		–	
Nafion-Ru OP/GCE*	SWV	G	0.84	1.1–10	2.2–33	0.006	0.076	Calf thymus DNA	–	^57^
		A	1.08	1.1–5	2.0–38	0.02	0.278		–	
BDDE/GCE*	DPV	G	1.15	0.21–23	0.3–19	0.037	0.158	Fish sperm	0.999	^58^
		A	1.35	0.12–25	0.3–19	0.019	0.067		0.999	
MWCNT/NiFe_2_O_4_/GCE*	LSV	G	0.73	0.05–3	0.1–40	0.006	0.012	ssDNA	0.002	^59^
		A	1.00	0.1–4	0.1–40	0.001	0.08		0.993	
Gr-Nafion/ GCE*	DPV	G	0.90	2–200	4–200	0.58	0.58	Herring sperm DNA	–	^60^
		A	1.26	5–200	8–150	0.75	0.75		–	
β-Cyclodextrin/MWNTs^@^	DPV	G	0.63	100—280	–	0.75 nM	–	Salmon sperm DNA	–	^61^
		A	0.90	4.0–20.0	–	6.76 nM	–		–	
		T	1.10	80–400	–	33.67 Nm	–			
NiCNF/GCE*	DPV	G	0.80	0.05–2	0.05–2	0.03	0.03	Fish sperm	0.999	^62^
		A	1.10	0.05–2	0.05–2	0.03	0.03		0.999	
TAN/AgNP/PANF/CPE*	DPV	G	0.6	0–15	0–200	24.0	3.0	Fish sperm	0.991	^63^
		A	1.0	0–25	0–140	0.06	2.8		0.998	
Nafion/g-C_3_N_4_/GNF/GCE*	DPV			0.6 × 10^–6^–7.4 × 10^–6^	0.3 × 10^–6^–6.6 × 10^–6^	0.0047	0.0069		0.999	
		G	0.62	0.3 × 10^–6^–5.3 × 10^–6^	0.6 × 10^–6^–7.6 × 10^–6^	0.0035	0.0064		0.995	
		A	1.00	6.6 × 10^–6^–60 × 10^–5^	5.3 × 10^–6^–63.3 × 10^–6^	0.055	0.067	Chicken liver and beef liver	0.999	This work
	AMP	T	1.30	0.6 × 10^–6^ – 12.6 × 10^–6^		0.0046			0.9983	
				0.3 × 10^–6^ – 12.6 × 10^–6^		0.0013			0.9986	
				5.6 × 10^–6^ – 50.6 × 10^–4^		0.051			0.9992	

^@^ represent the individual analyte measurements whereas * indicates simultaneous analysis of nucleic acid bases.

DPV technique was employed for concurrent detection of DNA bases, due to its excellent improved resolution and excellent current sensitivity in contrast with CV. The concurrent determination of DNA bases was also conceded out by means of Nafion/g-C_3_N_4_/GNF/GCE in the existence of buffer neutral pH. In Fig. 7D, the linear increase in peak current for each analyte with increased concentration of DNA bases and the peak oxidation potential persist without any changes. The suggested DPV technique is capable of detecting DNA bases concurrently and sensitively exclusive of critically interfering with one another. DPV studies defined the oxidation peak current response for ternary combination of G, A, and T are well alienated one another along with a difference in potentials of 0.148 V, 0.129 V and 0.177 V. The linear relationship between the DNA bases concentrations are ranging from 0.3 × 10^–7^ to 6.6 × 10^–6^ M for ‘G’ (inset), 0.3 × 10^–7^ to 7.3 × 10^–6^ M for ‘A’, and 5.3 × 10^–6^ to 63.3 × 10^–4^ for ‘T’ (Fig. 7E, F).

It is observed that DNA bases can be successfully detected from its mixture at different concentrations range. We could see the proposed consequences as well as analytical parameters inclusive of concentrations in the linear range, pH, regression coefficient, limit of the detection and linear regression equation for the detection of ternary mixtures (Table S1). Additionally, the proposed electrode system is quite simple and stable with lower detection limits, and easier to prepare. The sensor can be used with individual nucleic acid bases (G, A and T) and simultaneous detection of all nucleic acid bases in a single run of DPV analysis.

### Amperometry findings

**Figure 8:**
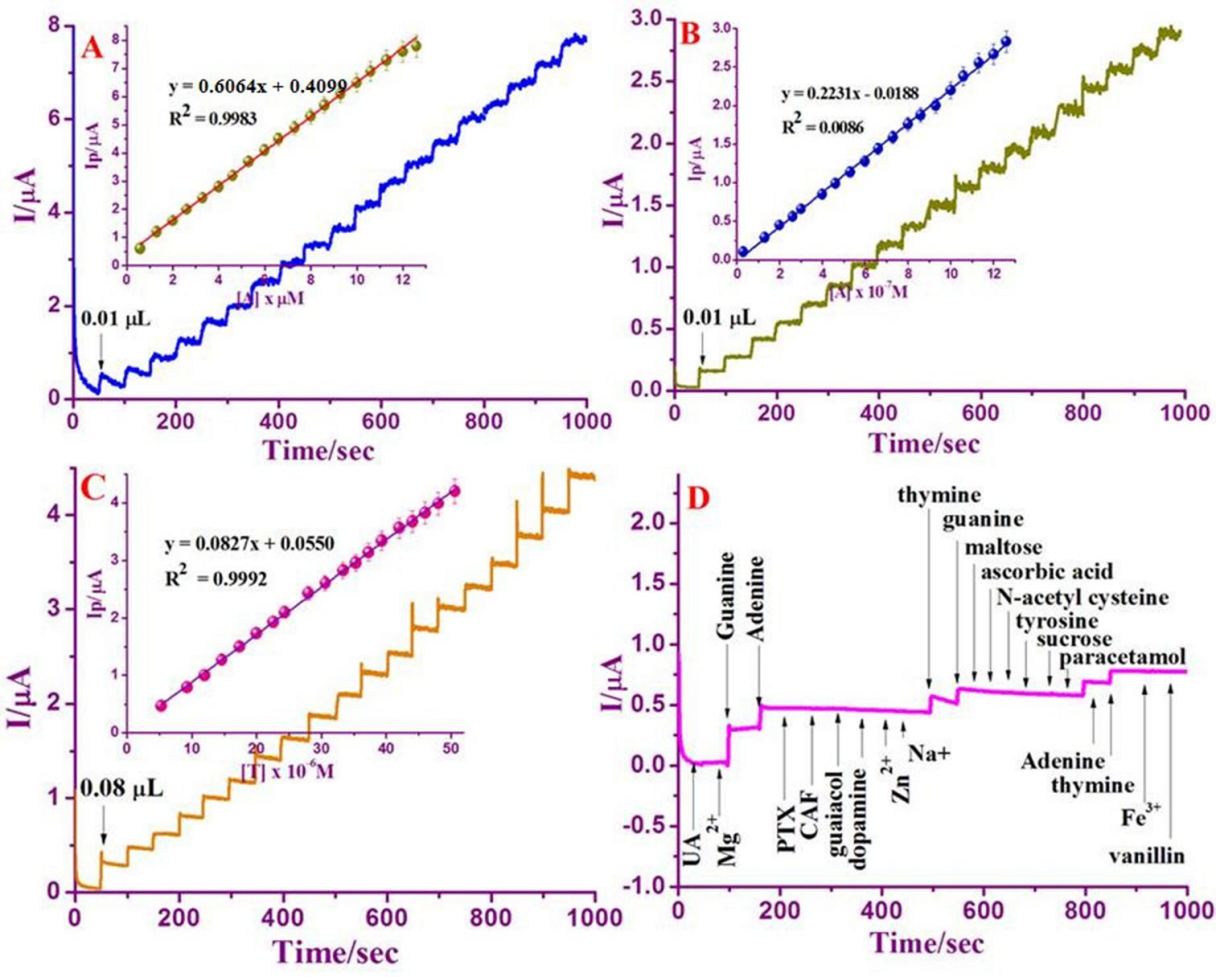
Amperometric response at Nafion/g-C_3_N_4_/GNF/GCE with an applied potential + 0.8 V, + 1.0 V, and + 1.3 to the following addition of various concentrations from of 0.6 × 10^–6^ M to 12.6 × 10^–5^ M of ‘G’ (**A**), 0.6 × 10^–6^ M to 12.6 × 10^–5^ M of ‘A’ (**B**), and 5.3 × 10^–6^ to 50.6 × 10^–5^ M for T (**C**) in the existence of 0.1 M buffer (neutral pH). Amperometric response of some interfering compounds at Nafion/g-C_3_N_4_/GNF/GCE in the existence of 5 μM of ‘G’, 5 μM of and 30 μM of T via 0.1 M buffer (neutral pH) (**D**).

Amperometry can be used to assess the current signal for each addition of DNA bases under agitation. The characteristic steady-state catalytic current–time (i–t) using Nafion/g-C_3_N_4_/GNF-modified GCE under the mild stirring condition for the dropping of 1 μM ‘G’, 1 μM ‘A’ and 5 μM ‘T’ for 50 s with 0.1 M buffer (neutral pH) corresponding to the supplied voltage of + 0.8 V, + 1.0 V and + 1.3 V against Ag/AgCl electrode. Every addition of DNA base for 50 s intervals resulting increase in current signal thereafter the steady-state current was attained within 5 s. The rapid current signal suggests that Nafion/g-C_3_N_4_/GNF/GCE has shown its excellence oxidizing of DNA bases, which is appropriate for the practical applications. From Fig. 8A–C, it is apparent that the oxidative current rises with increasing in concentration of DNA bases. Additionally, amperometric response also increases with a linear concentration in the range of 0.6 × 10^–6^ M to 12.6 × 10^–5^ M for ‘G’ and 0.6 × 10^–6^ M to 12.6 × 10^–5^ M for ‘A’ and 5.3 × 10^–6^ to 50.6 × 10^–5^ M for ‘T’. A calibration plot attained with the coefficient of correlation for G, A, and T are 0.9983, 0.9986 and 0.9992, respectively (Inset: Fig. 8A–C), which justify the good correlation between oxidative peak current and DNA bases concentration. Detection limit was determined by calibrating G, A and T, and were signified to be 4.6 nM, 13 nM, and 51 nM, and this is can be compared with previously published results (Table 1), which indicates that Nafion/g-C_3_N_4_/GNF / GCE is ideally suited for DNA bases detection.

### Interference

The influence of electroactive interferences on detection of DNA bases can be examined by the addition of foreign species into the buffer (neutral pH) having 5 μM of ‘G’, 5 μM of ‘A’ and 30 μM of ‘T’. This outcome signified that widespread inorganic ions, for instance, a 100 fold excess of Zn^2+^, Fe^2+^, Mg^2+^, and Na^+^ had almost no interference on the determination. 50-fold concentrations of pentaoxifylline, paracetamol, trysoine: 20-fold concentrations of caffeine, guaiacol, sucrose, and maltose hardly impact the peak signals of the bases. Dopamine, uric acid, ascorbic acid, *N*-acetyl cysteine and vanillin, which run in parallel within the biological molecules of DNA bases, have minor effects, however it is insignificant (Fig. 8D). Thus, the presented biosensor highly recommended in the real sample analysis to examine the content of DNA bases.

### Reproducibility and electrochemical stability

The repeatability of the analytical signal is being explored. To determine the proficiency of an electrochemical biosensor, a sequence of monotonous voltammetry measurements was done at the Nafion/g-C_3_N_4_/GNF-modified GCE. The RSD for the five successive determinations of 5 μM of ‘G’, 5 μM of ‘A’, and 30 μM of ‘T’ were 2.0%, 2.8% and 3.3%, respectively. The stability of Nafion/g-C_3_N_4_/GNF/GCE for prolonged period was investigated. The concentration of DNA bases such as 5 μM of ‘G’, 5 μM of ‘A’, and 30 μM of ‘T’ was measured and there is no considerable reduction in oxidation peak signal was noticed and after 4 days of measurement only ~ 3% decrement of peak intensity was noted after 30 days. Hence, the storage stability of the designed electrode is important characteristics for the persistent operations.

### Real sample analysis

Under the most favorable conditions, the practical application of Nafion/g-C_3_N_4_/GNF/GCE was determined by electrochemical evaluation of G, A, and T with Chicken liver and Beef liver samples. Proceeding to the evaluations, the real specimens were pretreated as labeled in experimentally and then diluted with DI water. The analytical consequences were scheduled in Table 2. The achieved results yield the excellent recoveries in Chicken liver in the ranges of 99%, 99.1% and, 100.7% for ‘G’, 99.3%, 102.6% , and 103.7% for ‘A’ and 101.4%, 102.06%, and 105.2% for ‘T’, respectively, In Beef liver, 101.1% , 101.8%, and 105.2% for ‘G’, 96.7%, 98.1% , and 101.8% for ‘A’ and 97.6%, 101.1%, and 107.7% for ‘T’, respectively. Moreover, its RSD estimation for particle sample evaluation was lower than 4% and is within the appropriate range. The entire results exposed that the designed electrode is practicable and can be applicable for the concurrent valuation of G, A, and T in the actual biological specimens.

**Table 2:** Assay results for the DNA bases in chicken liver and beef liver (n = 3).

Sample	Added (µg mL^−1^)			Found (µg mL^−1^)			Recovery (%)			Mean ± SD (%)		
	G	A	T	G	A	T	G	A	T	G	A	T
Chicken liver	2	3	10	1.98	2.98	10.14	99	99.3	101.4	1.2	2.0	2.8
	6	5	15	5.95	5.13	15.39	99.1	102.6	102.6	2.4	2.2	3.5
	7	9	20	7.05	9.34	21.05	100.7	103.7	105.2	3.0	2.9	4.0
Beef liver	1	4	15	1.01	3.87	14.65	101.1	96.7	97.6	2.5	2.7	2.5
	5	7	30	5.09	6.87	30.35	101.8	98.1	101.1	2.9	3.4	3.4
	10	12	50	10.52	12.22	53.88	105.2	101.8	107.7	3.6	3.9	4.0

## Conclusion

This research established a novel nanocomposite material for the simultaneous finding of nucleobases such by DPV method at trace level. It is proposed that a novel sensing nanocomposite which consisting of *π–π* stacking interactions co-existing of g-C_3_N_4_ and GNF provide a stable platform with high electronic conductivity developed by a simple hydrothermal method. This hybrid nanocomposite has some advantages over the other composite systems including elimination of surface fouling and the enhanced edge defects with enhance electrocatalytic oxidation behavior. These are the important salient feature for the proposed sensing device. The Nafion/g-C_3_N_4_/GNF/GCE were employed for individual evaluation of DNA bases with the detection limits of 4.5 nM, 3.5 nM, and 55 nM, respectively. Moreover, the recommended sensor with nanocomposite modified electrode proven for excellence in concurrent quantitative assessment of nucleobases in actual samples.

## Supplementary information

Supplementary Information.

## Data Availability

Relevant data is available in the Supplementary Source file.
