# Supplementary material for: Graphitic carbon nitride/graphene nanoflakes hybrid system for electrochemical sensing of DNA bases in meat samples
Source: Sci Rep. 2020 Jul 30;10:12860. doi: 10.1038/s41598-020-69578-8 (PMC7393070; doi:10.1038/s41598-020-69578-8)
Supplement: Supplementary file 1 — Supplementary Information. [file 41598_2020_69578_MOESM1_ESM.pdf]

Supporting Information

Graphitic Carbon Nitride/ Graphene Nanoflakes Hybrid System for Electrochemical Sensing of DNA Bases in Meat Samples

J. Kalaiyarasi[a], K. Pandian[a*], Santheraleka Ramanathan[b],

Subash C.B. Gopinath[b,c]**

[a]Department of Inorganic Chemistry, University of Madras,
Guindy campus, Chennai - 600 025, India
[b]Institute of Nano Electronic Engineering, 01000 Kangar, & [c]School of Bioprocess Engineering, 02600 Arau, Universiti of Malaysia Perlis, Perlis, Malaysia.

To whom address correspondence should be made:

Prof. K. Pandian,
Email: jeevapandian@yahoo.co.uk or subash@unimap.edu.my
Tel.: +91-44-22202795

Figure S1

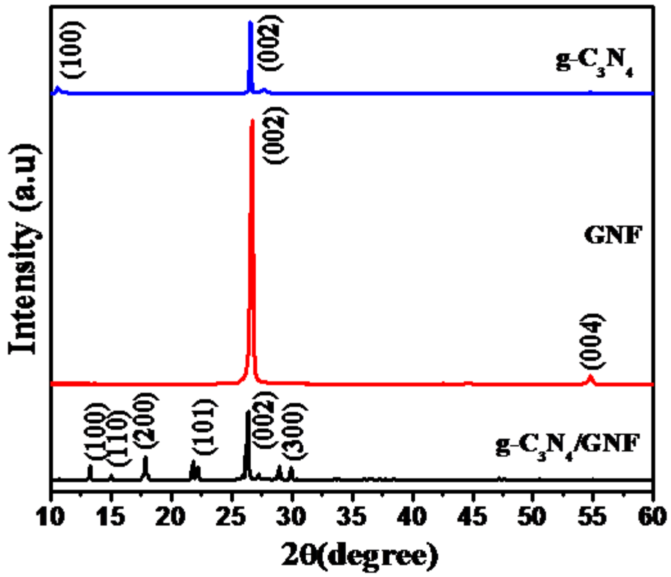

Fig.S1 XRD pattern of g-C_3N_4, GNF, andg-C_3N_4/GNF nanocomposites.

Figure S2

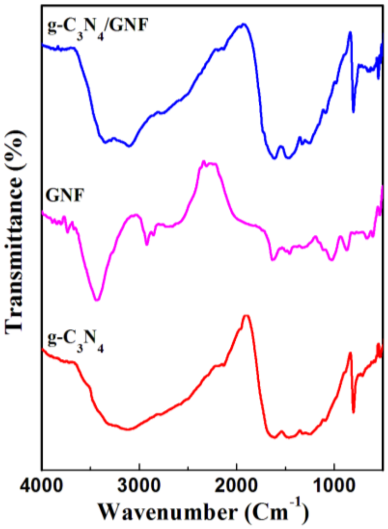

Fig.S2 FT-IR spectra of a) g-C_3N_4, b) GNF and c)g-C_3N_4/GNF nanocomposites.

Figure S3

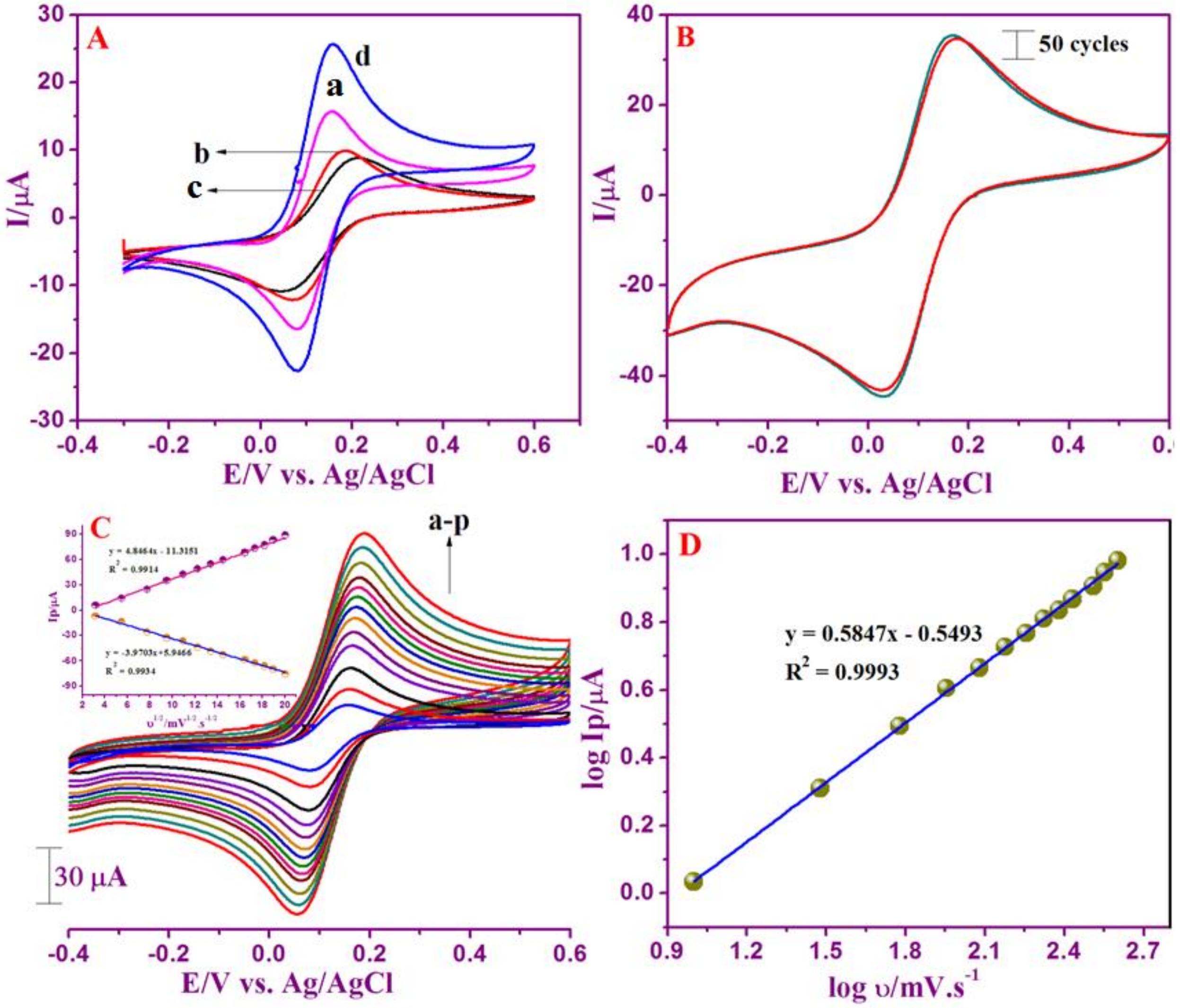

Figure S3: ***Cyclic voltammograms of, a) bare GCE, b) GNF/GCE, c) g-C_3N_4/GCE and d) Nafion®/g-C_3N_4/GNF/GCE in 10mM $[Fe(CN)_6]^{3-/4-}$having 0.1 M KCl. Scan rate: 50 mV/s (A). Cyclic voltammograms of Nafion®/g-C_3N_4/GNF/GCE in 10mM $[Fe(CN)_6]^{3-/4-}$having 0.1 M KCl at continuous 50 cycles (B). Cyclic voltammograms of Nafion®/g-C_3N_4/GNF/GCE in 0.1 M KCl along with 10 mM $[Fe(CN)_6]^{3-/4-}$ at different scan rates 10 – 400 mV.s^{-1}(C). Inset: Linear plot I_{pa} and I_{pc}vs.$\upsilon^{1/2}$. (D) Linear plot log $I_p$vs. logυ.***

Figure S4

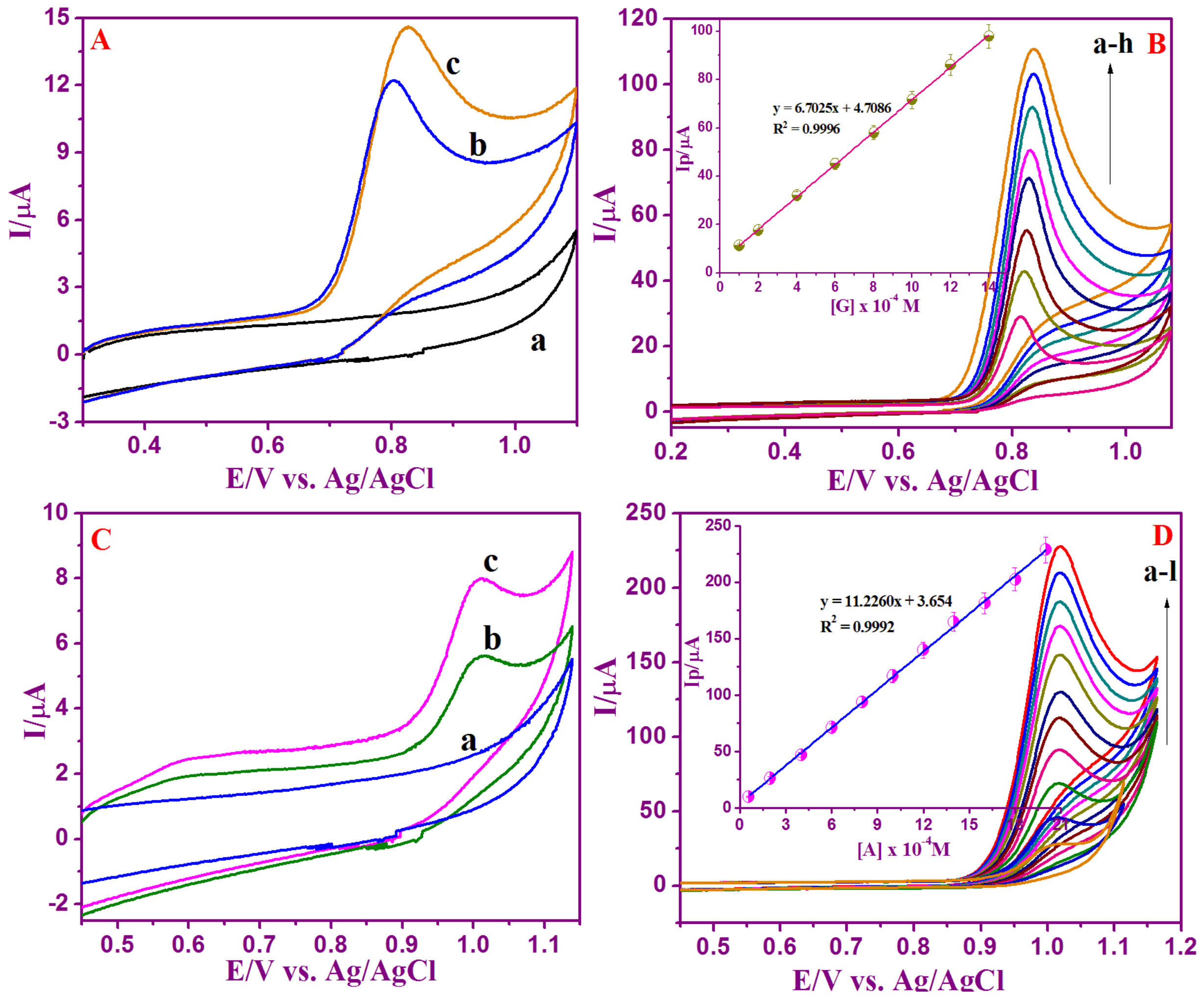

Figure S4: ***CV behaviour of a) bare GCE, b)bare GCE in the existence of 3.3 × 10^{-4}M of G, and c) Nafion®/g-C_3N_4/GNF/GCE in the existence of 3.3 × 10^{-4}M of G and at scan rate of 50 mV.s^{-1} in 0.1 M KCl having PBS with pH 7.0 (A). Cyclic voltammograms of Nafion®/g-C_3N_4/GNF/GCE in various concentrations of 'G' from 1 x 10^{-4} to 14 x 10^{-3} M in 0.1 M KCl having PBS with pH 7.0. Inset: Calibration plot of I_{pa}vs. Conc. of 'G' (B). CV behaviour of a) bare GCE, b)bare GCE in the existence of 3.3 × 10^{-4}M of 'A', and c) Nafion®/g-C_3N_4/GNF/GCE in the existence of 3.3 × 10^{-4}M of 'A' at a scan rate of 50mV/s in 0.1 M KCl having PBS with pH 7.0 (C).Cyclic voltammograms of g-Nafion®/g-C_3N_4/GNF/GCE in dissimilar concentrations of adenine as 0.6 × 10^{-6}***

to 20× 10⁻ ^{5}M in 0.1 M KCl having PBS with pH 7.0. Inset: Calibration plot I_{pa}vs. Conc. of ‘A’ (D).

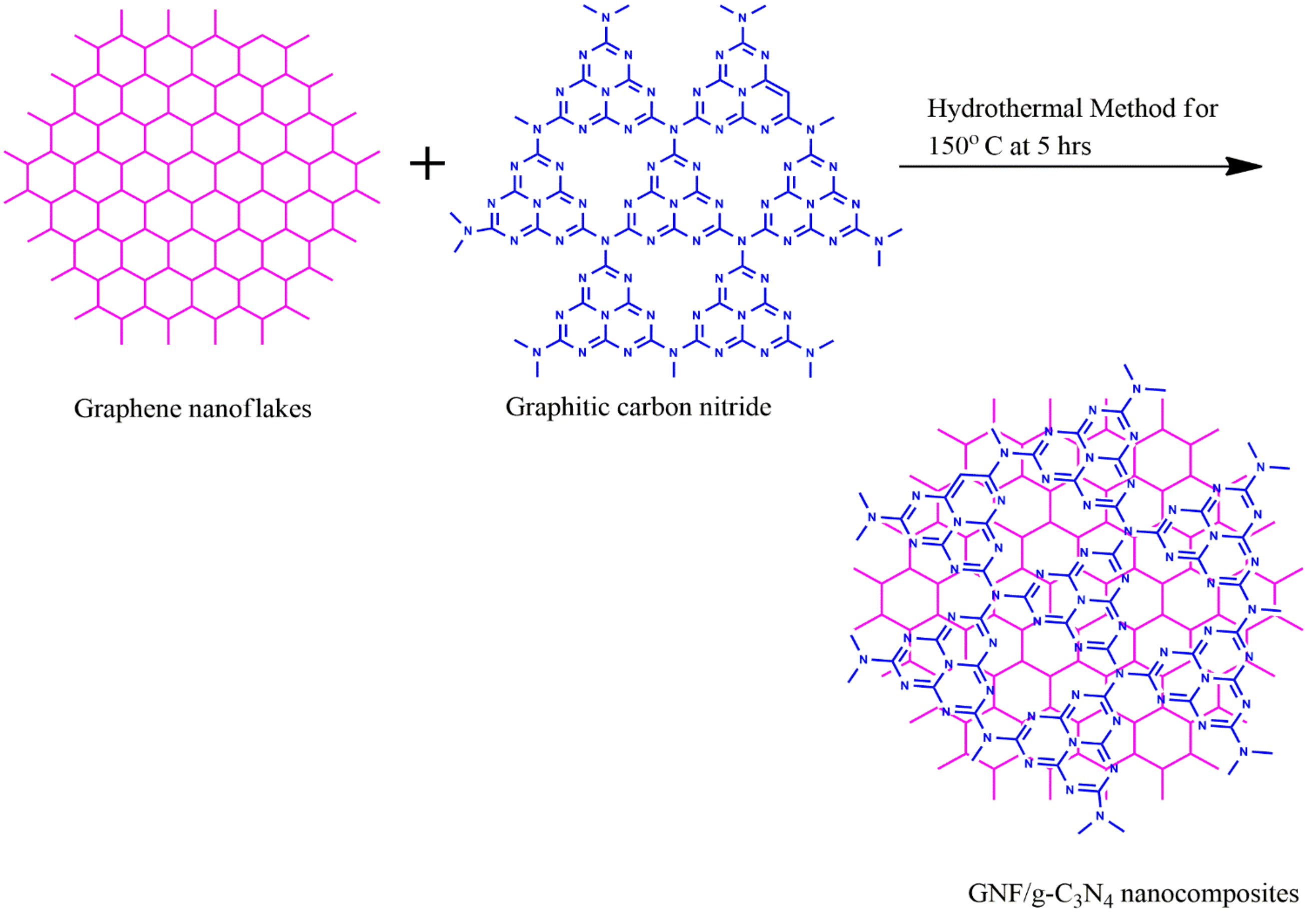

SchemeS1 : Schematic diagram for preparation of GNF/g-C_3N_4nanocomposites

Table S1: Linear regression analysis and detection limit using DPV for the quantification of an and simultaneous of G, A and T

Process	DNA bases	Linear regression equation	R^2	Linear range (μM)	LOD (μM)
**Individual**	G	Ip=6.3748x+0.7313	0.9974	0.6×10^{-6}- 7.4×10^{-6}	0.0047
	A	Ip=8.4811x+0.0370	0.9987	0.3×10^{-7}- 5.3×10^{-6}	0.0035
	T	Ip=0.5371x-0.1745	0.9930	6.6×10^{-6} – 60.0×10^{-4}	0.055
**Simultaneous**	G	Ip=4.3466x+1.0636	0.9992	0.3×10^{-7}- 6.6×10^{-6}	0.0069
	A	Ip=4.6379x+1.8600	0.9953	0.3×10^{-7}- 7.3×10^{-6}	0.0064
	T	Ip=0.4148x+1.1189	0.9993	5.3×10^{-6} - 63.3×10^{-4}	0.067
